# 2-(2*H*-Tetra­zol-5-yl)pyridinium chloride

**DOI:** 10.1107/S1600536808032649

**Published:** 2008-10-15

**Authors:** Jing Dai, Xiao-Chun Wen

**Affiliations:** a Ordered Matter Science Research Center, College of Chemistry and Chemical Engineering, Southeast University, Nanjing 210096, People’s Republic of China

## Abstract

In the title compound, C_6_H_6_N_5_^+^·Cl^−^, the pyridinium and tetra­zole rings are essentially coplanar. The pyridine N atoms are protonated. In the crystal structure, mol­ecules are connected *via* N—H⋯Cl, C—H⋯Cl, C—H⋯N and N—H⋯N hydrogen bonds into layers that are parallel to the (001) plane. There are two crystallographically independent mol­ecules in the asymmetric unit which are located on mirror planes.

## Related literature

For related literature on tetra­zole derivatives, see: Dai & Fu (2008 ▶); Wang *et al.* (2005 ▶); Wen (2008 ▶); Xiong *et al.* (2002 ▶).
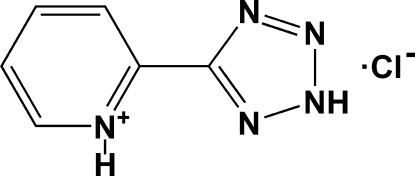


## Experimental

### 

#### Crystal data

C_6_H_6_N_5_^+^·Cl^−^
*M* *_r_* = 183.61
Orthorhombic, 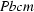

*a* = 16.375 (3) Å
*b* = 15.313 (3) Å
*c* = 6.5176 (13) Å
*V* = 1634.3 (5) Å^3^
*Z* = 8
Mo *K*α radiation
μ = 0.41 mm^−1^
*T* = 298 (2) K
0.25 × 0.20 × 0.18 mm

#### Data collection

Rigaku Mercury2 diffractometer
Absorption correction: multi-scan (*CrystalClear*; Rigaku, 2005 ▶) *T*_min_ = 0.910, *T*_max_ = 0.938
16127 measured reflections
2041 independent reflections
1511 reflections with *I* > 2σ(*I*)
*R*_int_ = 0.076

#### Refinement

*R*[*F* ^2^ > 2σ(*F* ^2^)] = 0.062
*wR*(*F* ^2^) = 0.148
*S* = 1.14
2041 reflections
145 parameters
H-atom parameters constrained
Δρ_max_ = 0.32 e Å^−3^
Δρ_min_ = −0.23 e Å^−3^

### 

Data collection: *CrystalClear* (Rigaku, 2005 ▶); cell refinement: *CrystalClear*; data reduction: *CrystalClear*; program(s) used to solve structure: *SHELXS97* (Sheldrick, 2008 ▶); program(s) used to refine structure: *SHELXL97* (Sheldrick, 2008 ▶); molecular graphics: *SHELXTL* (Sheldrick, 2008 ▶); software used to prepare material for publication: *SHELXTL*.

## Supplementary Material

Crystal structure: contains datablocks I, global. DOI: 10.1107/S1600536808032649/nc2115sup1.cif

Structure factors: contains datablocks I. DOI: 10.1107/S1600536808032649/nc2115Isup2.hkl

Additional supplementary materials:  crystallographic information; 3D view; checkCIF report

## Figures and Tables

**Table 1:** Hydrogen-bond geometry (Å, °)

*D*—H⋯*A*	*D*—H	H⋯*A*	*D*⋯*A*	*D*—H⋯*A*
N9—H9*A*⋯Cl2	0.86	2.14	3.001 (3)	177
N10—H10*A*⋯Cl1	0.86	2.33	3.088 (3)	147
N2—H2⋯Cl2	0.86	2.22	3.050 (4)	163
N5—H5*A*⋯Cl1^i^	0.86	2.29	3.049 (3)	147
N5—H5*A*⋯N1	0.86	2.55	2.881 (4)	104
N10—H10*A*⋯N6	0.86	2.52	2.858 (4)	105
C9—H9⋯Cl2	0.93	2.64	3.545 (4)	165
C3—H3⋯N8^ii^	0.93	2.60	3.329 (5)	136
C6—H6⋯N6^i^	0.93	2.38	3.260 (5)	159
C10—H10⋯Cl1^iii^	0.93	2.67	3.596 (4)	174

Symmetry codes: (i) 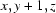; (ii) 
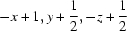
; (iii) 
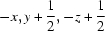
.

**Fig. 1.**
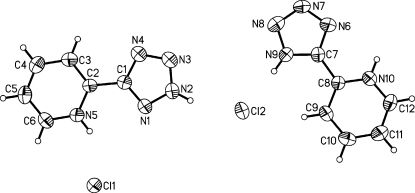
A view of the title compound with the atomic numbering scheme. Displacement ellipsoids were drawn at the 30% probability level.

**Fig. 2.**
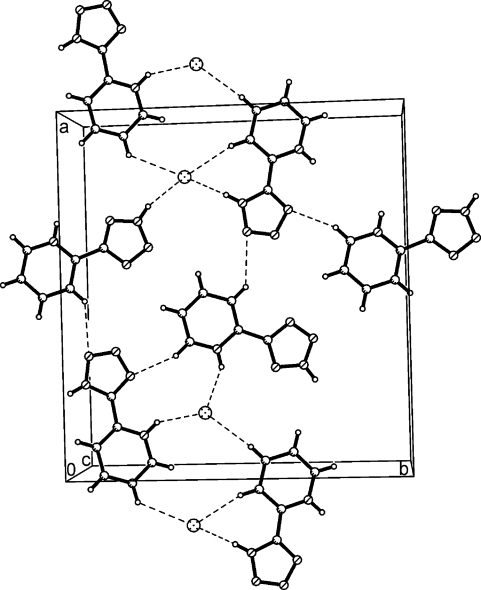
The crystal packing of the title compound viewed along the c axis showing the two-dimensionnal hydrogen bondings network.

## supplementary crystallographic information

### Comment

Tetrazole derivatives have found wide range of applications in coordination chemistry because of their multiple coordination modes as ligands to metal ions and for the construction of novel metal-organic frameworks (Wang *et al.*, 2005; Xiong *et al.*, 2002; Wen, 2008). In our ongoing investigations in this field we report here the crystal of 2-(2*H*-tetrazol-5-yl)pyridine-1-ium chloride (Fig.1).

In the crystal structure there are two crystallographically independent molecules, both of them located on mirror planes. Therefore, the benzene and tetrazole rings in both independent molecules are essentially planar. The geometric parameters of the tetrazole rings are comparable to those in related molecules (Wang *et al.*, 2005; Dai & Fu, 2008).

The crystal structure is stabilized by N—H···Cl, C—H···Cl, C—H···N and N—H···N hydrogen bonding. The different H bonding interactions connect the molecules into layers, that are parallel to the (0 0 1) plane (Table 1, Fig. 2).

### Experimental

Picolinonitrile (30 mmol), NaN _3_ (45 mmol), NH_4_Cl (33 mmol) and DMF (50 ml) were added in a flask under nitrogen atmosphere and the mixture stirred at 110°C for 20 h. The resulting solution was then poured into ice-water (100 ml), and a white solid was obtained after adding HCl (6 *M*) till pH=6. The precipitate was filtered and washed with distilled water. Colourless block-shaped crystals suitable for X-ray analysis were obtained from the crude product by slow evaporation of the solvent from an ethanol/HCl (50:1 *v*/*v*) solution.

### Refinement

All H atoms were located in difference map but were positioned with idealized geometry with C—H = 0.93 Å and N—H = 0.86 Å and were refined isotropic with *U*_iso_(H) = 1.2Ueq(C or N) using a riding model.

### Crystal data

**Table:**

C_6_H_6_N_5_^+^·Cl^−^	*F*(000) = 752
*M**_r_* = 183.61	*D*_x_ = 1.492 Mg m^−^^3^
Orthorhombic, *P**b**c**m*	Mo *K*α radiation, λ = 0.71073 Å
Hall symbol: -P 2c 2b	Cell parameters from 2986 reflections
*a* = 16.375 (3) Å	θ = 2.5–27.5°
*b* = 15.313 (3) Å	µ = 0.42 mm^−^^1^
*c* = 6.5176 (13) Å	*T* = 298 K
*V* = 1634.3 (5) Å^3^	Block, colourless
*Z* = 8	0.25 × 0.20 × 0.18 mm

### Data collection

**Table:**

Rigaku Mercury2 diffractometer	2041 independent reflections
Radiation source: fine-focus sealed tube	1511 reflections with *I* > 2σ(*I*)
graphite	*R*_int_ = 0.077
Detector resolution: 13.6612 pixels mm^-1^	θ_max_ = 27.5°, θ_min_ = 2.5°
ω scans	*h* = −21→21
Absorption correction: multi-scan (CrystalClear; Rigaku, 2005)	*k* = −19→19
*T*_min_ = 0.910, *T*_max_ = 0.938	*l* = −8→8
16127 measured reflections	

### Refinement

**Table:**

Refinement on *F*^2^	Primary atom site location: structure-invariant direct methods
Least-squares matrix: full	Secondary atom site location: difference Fourier map
*R*[*F*^2^ > 2σ(*F*^2^)] = 0.062	Hydrogen site location: inferred from neighbouring sites
*wR*(*F*^2^) = 0.148	H-atom parameters constrained
*S* = 1.14	*w* = 1/[σ^2^(*F*_o_^2^) + (0.0616*P*)^2^ + 0.4596*P*] where *P* = (*F*_o_^2^ + 2*F*_c_^2^)/3
2041 reflections	(Δ/σ)_max_ < 0.001
145 parameters	Δρ_max_ = 0.32 e Å^−^^3^
0 restraints	Δρ_min_ = −0.23 e Å^−^^3^

### Special details

**Table:**

Geometry. All e.s.d.'s (except the e.s.d. in the dihedral angle between two l.s. planes) are estimated using the full covariance matrix. The cell e.s.d.'s are taken into account individually in the estimation of e.s.d.'s in distances, angles and torsion angles; correlations between e.s.d.'s in cell parameters are only used when they are defined by crystal symmetry. An approximate (isotropic) treatment of cell e.s.d.'s is used for estimating e.s.d.'s involving l.s. planes.
Refinement. Refinement of *F*^2^ against ALL reflections. The weighted *R*-factor *wR* and goodness of fit *S* are based on *F*^2^, conventional *R*-factors *R* are based on *F*, with *F* set to zero for negative *F*^2^. The threshold expression of *F*^2^ > σ(*F*^2^) is used only for calculating *R*-factors(gt) *etc*. and is not relevant to the choice of reflections for refinement. *R*-factors based on *F*^2^ are statistically about twice as large as those based on *F*, and *R*- factors based on ALL data will be even larger.

### Fractional atomic coordinates and isotropic or equivalent isotropic displacement parameters (Å^2^)

**Table:**

	*x*	*y*	*z*	*U*_iso_*/*U*_eq_	
C9	0.0640 (2)	0.4651 (2)	0.2500	0.0494 (10)	
H9	0.0803	0.5232	0.2500	0.059*	
N6	0.26721 (19)	0.3543 (2)	0.2500	0.0476 (8)	
N7	0.33865 (17)	0.3973 (2)	0.2500	0.0466 (8)	
N8	0.3269 (2)	0.4819 (3)	0.2500	0.0568 (9)	
N9	0.24511 (18)	0.4938 (2)	0.2500	0.0480 (8)	
H9A	0.2202	0.5432	0.2500	0.058*	
C7	0.2096 (2)	0.4149 (2)	0.2500	0.0399 (8)	
C8	0.1215 (2)	0.3988 (2)	0.2500	0.0373 (8)	
N10	0.09641 (17)	0.31585 (19)	0.2500	0.0422 (8)	
H10A	0.1325	0.2750	0.2500	0.051*	
C12	0.0174 (2)	0.2938 (3)	0.2500	0.0516 (10)	
H12	0.0025	0.2352	0.2500	0.062*	
C11	−0.0412 (3)	0.3566 (3)	0.2500	0.0619 (12)	
H11	−0.0962	0.3414	0.2500	0.074*	
C10	−0.0181 (2)	0.4434 (3)	0.2500	0.0566 (11)	
H10	−0.0575	0.4871	0.2500	0.068*	
N4	0.4116 (2)	0.8572 (2)	0.2500	0.0671 (11)	
N3	0.3635 (2)	0.7880 (2)	0.2500	0.0663 (10)	
N2	0.2886 (2)	0.8188 (2)	0.2500	0.0615 (10)	
H2	0.2462	0.7856	0.2500	0.074*	
N1	0.2836 (2)	0.9048 (2)	0.2500	0.0591 (10)	
N5	0.33937 (17)	1.0833 (2)	0.2500	0.0414 (7)	
H5A	0.2880	1.0714	0.2500	0.050*	
C1	0.3619 (2)	0.9270 (2)	0.2500	0.0422 (9)	
C2	0.3931 (2)	1.0162 (2)	0.2500	0.0373 (8)	
C3	0.4760 (2)	1.0352 (2)	0.2500	0.0475 (9)	
H3	0.5145	0.9905	0.2500	0.057*	
C4	0.5005 (2)	1.1221 (3)	0.2500	0.0533 (10)	
H4	0.5559	1.1355	0.2500	0.064*	
C5	0.4444 (3)	1.1881 (3)	0.2500	0.0546 (11)	
H5	0.4611	1.2461	0.2500	0.066*	
C6	0.3620 (2)	1.1670 (3)	0.2500	0.0486 (9)	
H6	0.3228	1.2110	0.2500	0.058*	
Cl1	0.15729 (5)	0.12495 (6)	0.2500	0.0456 (3)	
Cl2	0.16485 (7)	0.66996 (7)	0.2500	0.0764 (4)	

### Atomic displacement parameters (Å^2^)

**Table:**

	*U*^11^	*U*^22^	*U*^33^	*U*^12^	*U*^13^	*U*^23^
C9	0.048 (2)	0.0309 (19)	0.070 (3)	0.0017 (17)	0.000	0.000
N6	0.0410 (18)	0.0434 (19)	0.059 (2)	0.0073 (14)	0.000	0.000
N7	0.0324 (16)	0.052 (2)	0.055 (2)	0.0076 (14)	0.000	0.000
N8	0.0363 (17)	0.061 (2)	0.073 (3)	−0.0044 (15)	0.000	0.000
N9	0.0340 (16)	0.0395 (18)	0.070 (2)	−0.0027 (13)	0.000	0.000
C7	0.041 (2)	0.0356 (19)	0.043 (2)	−0.0021 (16)	0.000	0.000
C8	0.0345 (18)	0.0339 (18)	0.044 (2)	−0.0007 (14)	0.000	0.000
N10	0.0408 (17)	0.0346 (16)	0.051 (2)	0.0027 (13)	0.000	0.000
C12	0.043 (2)	0.043 (2)	0.069 (3)	−0.0116 (18)	0.000	0.000
C11	0.036 (2)	0.059 (3)	0.091 (4)	−0.0024 (19)	0.000	0.000
C10	0.044 (2)	0.047 (2)	0.078 (3)	0.0132 (19)	0.000	0.000
N4	0.047 (2)	0.040 (2)	0.114 (3)	0.0033 (16)	0.000	0.000
N3	0.053 (2)	0.0384 (18)	0.107 (3)	0.0054 (17)	0.000	0.000
N2	0.046 (2)	0.0358 (18)	0.103 (3)	−0.0036 (16)	0.000	0.000
N1	0.0431 (19)	0.0336 (18)	0.100 (3)	0.0011 (14)	0.000	0.000
N5	0.0351 (16)	0.0371 (16)	0.0520 (19)	0.0015 (13)	0.000	0.000
C1	0.0372 (19)	0.0374 (19)	0.052 (2)	0.0022 (16)	0.000	0.000
C2	0.0327 (18)	0.0386 (19)	0.041 (2)	0.0023 (15)	0.000	0.000
C3	0.038 (2)	0.047 (2)	0.058 (3)	0.0049 (17)	0.000	0.000
C4	0.043 (2)	0.053 (2)	0.064 (3)	−0.0091 (19)	0.000	0.000
C5	0.055 (3)	0.039 (2)	0.071 (3)	−0.0112 (19)	0.000	0.000
C6	0.052 (2)	0.035 (2)	0.059 (3)	0.0049 (17)	0.000	0.000
Cl1	0.0394 (5)	0.0403 (5)	0.0572 (6)	−0.0005 (4)	0.000	0.000
Cl2	0.0564 (7)	0.0340 (5)	0.1387 (12)	−0.0022 (5)	0.000	0.000

### Geometric parameters (Å, °)

**Table:**

C9—C8	1.384 (5)	N4—N3	1.320 (5)
C9—C10	1.385 (5)	N4—C1	1.343 (5)
C9—H9	0.9300	N3—N2	1.315 (4)
N6—C7	1.324 (5)	N2—N1	1.320 (4)
N6—N7	1.343 (4)	N2—H2	0.8600
N7—N8	1.309 (5)	N1—C1	1.326 (5)
N8—N9	1.352 (4)	N5—C6	1.335 (5)
N9—C7	1.340 (4)	N5—C2	1.351 (4)
N9—H9A	0.8600	N5—H5A	0.8600
C7—C8	1.464 (5)	C1—C2	1.459 (5)
C8—N10	1.334 (4)	C2—C3	1.389 (5)
N10—C12	1.337 (4)	C3—C4	1.390 (5)
N10—H10A	0.8600	C3—H3	0.9300
C12—C11	1.359 (6)	C4—C5	1.365 (5)
C12—H12	0.9300	C4—H4	0.9300
C11—C10	1.382 (6)	C5—C6	1.387 (5)
C11—H11	0.9300	C5—H5	0.9300
C10—H10	0.9300	C6—H6	0.9300
			
C8—C9—C10	119.0 (4)	N3—N4—C1	106.1 (3)
C8—C9—H9	120.5	N2—N3—N4	105.5 (3)
C10—C9—H9	120.5	N3—N2—N1	114.6 (3)
C7—N6—N7	106.0 (3)	N3—N2—H2	122.7
N8—N7—N6	111.0 (3)	N1—N2—H2	122.7
N7—N8—N9	106.2 (3)	N2—N1—C1	101.2 (3)
C7—N9—N8	108.0 (3)	C6—N5—C2	123.3 (3)
C7—N9—H9A	126.0	C6—N5—H5A	118.4
N8—N9—H9A	126.0	C2—N5—H5A	118.4
N6—C7—N9	108.9 (3)	N1—C1—N4	112.5 (3)
N6—C7—C8	125.7 (3)	N1—C1—C2	125.3 (3)
N9—C7—C8	125.4 (3)	N4—C1—C2	122.2 (3)
N10—C8—C9	119.3 (3)	N5—C2—C3	118.5 (3)
N10—C8—C7	117.6 (3)	N5—C2—C1	118.9 (3)
C9—C8—C7	123.1 (3)	C3—C2—C1	122.5 (3)
C8—N10—C12	122.6 (3)	C2—C3—C4	118.8 (3)
C8—N10—H10A	118.7	C2—C3—H3	120.6
C12—N10—H10A	118.7	C4—C3—H3	120.6
N10—C12—C11	120.3 (4)	C5—C4—C3	121.0 (4)
N10—C12—H12	119.8	C5—C4—H4	119.5
C11—C12—H12	119.8	C3—C4—H4	119.5
C12—C11—C10	119.1 (4)	C4—C5—C6	118.8 (4)
C12—C11—H11	120.4	C4—C5—H5	120.6
C10—C11—H11	120.4	C6—C5—H5	120.6
C11—C10—C9	119.8 (4)	N5—C6—C5	119.6 (4)
C11—C10—H10	120.1	N5—C6—H6	120.2
C9—C10—H10	120.1	C5—C6—H6	120.2

### Hydrogen-bond geometry (Å, °)

**Table:**

*D*—H···*A*	*D*—H	H···*A*	*D*···*A*	*D*—H···*A*
N9—H9A···Cl2	0.86	2.14	3.001 (3)	177
N10—H10A···Cl1	0.86	2.33	3.088 (3)	147
N2—H2···Cl2	0.86	2.22	3.050 (4)	163
N5—H5A···Cl1^i^	0.86	2.29	3.049 (3)	147
N5—H5A···N1	0.86	2.55	2.881 (4)	104
N10—H10A···N6	0.86	2.52	2.858 (4)	105
C9—H9···Cl2	0.93	2.64	3.545 (4)	165
C3—H3···N8^ii^	0.93	2.60	3.329 (5)	136
C6—H6···N6^i^	0.93	2.38	3.260 (5)	159
C10—H10···Cl1^iii^	0.93	2.67	3.596 (4)	174

Symmetry codes: (i) *x*, *y*+1, *z*; (ii) −*x*+1, *y*+1/2, −*z*+1/2; (iii) −*x*, *y*+1/2, −*z*+1/2.
